# Research Progress on Probiotics in Alleviating Cow’s Milk Allergy: A Review

**DOI:** 10.3390/foods14111879

**Published:** 2025-05-26

**Authors:** Yangze Guo, Jiali Chen, Yezhi Qu, Xilin Wu, Shengyi Zhang, Jiale Wang, Xiqing Yue, Zhenmin Liu, Aijun Xie, Mohan Li

**Affiliations:** 1 State Key Laboratory of Dairy Biotechnology, Shanghai Engineering Research Center of Dairy Biotechnology, Shanghai 200436, China; 2 College of Food Science, Shenyang Agricultural University, Shenyang 110866, China; 3 School of Life Sciences & Biotechnology, Shanghai Jiao Tong University, Shanghai 200240, China; 4 Department of Biological Systems Engineering, University of Wisconsin-Madison, Madison, WI 53706, USA

**Keywords:** probiotics, cow’s milk allergy, gut microbiota, oral tolerance

## Abstract

Cow’s milk allergy (CMA) is one of the most common food allergies in infancy, capable of triggering severe allergic reactions. Alterations in gut microbial composition and function may be closely related to the development of CMA. Probiotics, as a means to modulate the gut microbial composition, demonstrate potential in controlling allergic reactions by enhancing gut barrier functions, promoting immune responses in the intestinal mucosa, and degrading potential allergens. Therefore, probiotics are increasingly considered a promising strategy for managing CMA. This review summarizes the major allergens in cow’s milk, their mechanisms of allergenicity, and the role and mechanisms of probiotics in alleviating CMA. It aims to provide a deeper understanding and fresh perspectives to support the use of probiotics as an effective approach for the prevention and treatment of CMA, encouraging broader clinical application and research.

## 1. Introduction

Food allergy is a specific immune response that is harmful to health and occurs repeatedly after ingesting certain foods, which has become a major global public health concern [1,2,3]. Among the eight major food allergens identified by the Food and Agriculture Organization (FAO), milk and dairy products (including lactose) are classified as significant allergens [4]. Cow’s milk is the third most prevalent food allergen in pediatric and mixed-age populations, following peanut and tree nut allergies [5]. Correspondingly, cow’s milk allergy (CMA) ranks among the most prevalent food allergies in infancy and is one of the top three causes of severe allergic reactions in children, with reported prevalence rates of 8–15% [6,7,8]. CMA is an abnormal immune response triggered by allergens in cow’s milk, typically manifesting as eczema, respiratory symptoms, gastrointestinal disturbances, and other related conditions. It is closely related to immunoglobulin E (IgE)- and immunoglobulin G (IgG)-mediated hypersensitivity reactions [9].

Probiotics, as defined by the FAO and World Health Organization (WHO) in 2001, are live microorganisms that confer health benefits to the host when administered in adequate amounts [10,11,12]. A key characteristic of probiotics is their ability to promote health, which must be demonstrated through scientifically validated strains with specific functions and efficacy [13,14,15]. Various probiotic species and strains have been reported to exhibit beneficial effects, including *Saccharomyces boulardii*, *Lacticaseibacillus casei*, *Bifidobacterium animalis* subsp. *lactis*, *Lacticaseibacillus rhamnosus*, *Bifidobacterium infantis*, *Lactiplantibacillus plantarum*, *Lactobacillus acidophilus*, and *Lacticaseibacillus paracasei* [16,17,18,19,20,21,22]. Many studies have indicated that probiotics can alleviate allergic reactions by modulating T helper 1 cells (Th1)/T helper 2 cells (Th2) and T helper 17 cells (Th17)/regulatory T cell (Treg) immune balance, restoring gut microbiota homeostasis, antagonizing pathogens, enhancing intestinal mucosal barrier integrity, and regulating immune function through enzymatic activity and beneficial metabolites [23,24,25,26].

Herein, this review aims to summarize the major allergens in cow’s milk, their allergic mechanisms, and the effects and mechanisms of probiotics in alleviating CMA, providing a theoretical foundation for precision nutrition using probiotics.

## 2. Major Allergens in Cow’s Milk

Food allergens are components in food that selectively activate immune cells and trigger abnormal immune responses, with most being proteins. Cow’s milk proteins constitute 3–5% of total milk content, with caseins comprising 80–85% and whey proteins 15–20% [27]. Some milk proteins contain linear or conformational antigenic epitopes recognized by the human immune system. Upon ingestion, these proteins may be identified as “harmful” substances, stimulating immune cells to produce antibodies, primarily IgE [28]. Excessive IgE binds to mast cells and other receptor-bearing cells. Upon re-exposure to the antigen, these cells release histamines and other mediators, leading to allergic reactions. The most common allergenic proteins in cow’s milk are caseins, β-lactoglobulin (β-LG), and α-lactalbumin (α-LA) [29], which differ significantly in composition and concentration from those in human milk.

### 2.1. Caseins

Caseins are a family of calcium-binding phosphoproteins found in milk in micellar form, accounting for 80–85% of total milk protein [30]. They are encoded by different genes located on the same chromosome and classified into αs1-, αs2-, β-, and κ-caseins. Differences in structure and content between human and bovine caseins contribute to their allergenicity.

A study indicated that approximately 65% of individuals with milk allergy react to caseins [31]. Bernard et al. isolated and purified four types of caseins and conducted antigen-antibody reactions by immobilizing these antigens with sera from 58 children allergic to total casein [32]. Their findings revealed that 85% of these children exhibited specific IgE reactivity to all four caseins. The amino acid composition of these four caseins has been extensively studied. Thus, the identification of IgE-binding epitopes has become a key strategy in the investigation of milk protein allergy. Numerous studies have mapped IgE-binding regions within milk protein amino acid sequences and further identified key residues involved in IgE binding. Spuergin et al., using synthetic peptide methodologies, identified three IgE-binding regions in the αs1-casein sequence that triggered allergic responses in sensitized individuals: positions 19–30, 93–98, and 141–150 [33]. These sequences were located within the hydrophobic region of casein, suggesting that denaturation or degradation of casein in the human body could enhance its allergenicity. Chatchatee et al. employed cellulose-derivative membrane synthesis and identified six major IgE-binding epitopes on αs1-casein [34], including the three sequences previously identified by Spuergin et al. Their study also highlighted differences in epitope recognition between individuals with persistent and transient CMA. Similarly, Jarvinen et al. synthesized known IgE-binding peptides from milk proteins on cellulose membranes and found that five casein-derived peptides were recognized by sera from milk-allergic patients [35]. Furthermore, Cerecedo et al., using peptide chip immunoassays, studied the specificity of IgE and IgG antibodies binding to milk proteins and identified eight highly allergenic peptide fragments among the four caseins [36]. Their findings indicated that the recognition patterns of these milk proteins differed between allergic patients and healthy individuals. Cong et al. applied serological methods to determine IgE and IgG epitopes in αs1-casein and employed alanine-scanning mutagenesis to pinpoint critical allergenic residues at positions 22 and 23 of αs1-casein [37].

Although these studies employed different methodologies and thus identified varying allergenic fragments, several key antigenic epitopes have been consistently confirmed across multiple studies. These include residues 69–178 and 173–194 in αs1-casein, residues 171–180 and 191–200 in αs2-casein, residues 45–50, 55–70, and 173–194 in β-casein, and residues 13–22, 34–44, and 155–164 in κ-casein.

### 2.2. Whey Proteins

α-LA and β-LG are the main sensitizing components of whey proteins [38]. Compared to caseins, whey proteins have a higher degree of secondary and tertiary structures, and β-LG also exhibits a quaternary structure [39,40]. These proteins are resistant to acid and enzymatic hydrolysis due to their lack of phosphorylation and the presence of intramolecular disulfide bonds, allowing them to retain structural integrity during digestion and trigger immune responses [41]. Studies have shown that among individuals with CMA, approximately 27.6–62.8% exhibit sensitivity to α-LA, while approximately 82% are allergic to β-LG [36,42,43]. In a study conducted by Shi et al. on the antibody specificity of major milk allergens in the sera of milk-allergic children in China, the IgE positivity rates for α-LA and β-LG were found to be 44.3% and 39.3%, respectively [44].

Regarding the allergenic peptide segments of whey proteins, Jarvinen et al. utilized a cellulose-derived membrane synthesis method to analyze the recognition patterns of α-LA and β-LG in the sera of 11 CMA patients [35]. Their findings identified four IgE-binding regions and three IgG-binding regions on α-LA, as well as seven IgE-binding regions and six IgG-binding regions on β-LG. Hochwarner et al. reported that among 66 CMA patients, 57.6% exhibited IgE reactivity to α-LA, and they identified six IgE-binding regions on α-LA [45], three of which overlapped with those previously identified by Jarvinen et al. Similarly, Li et al. identified six linear IgE-binding epitopes on α-LA, two of which were consistent with the findings of prior studies [46]. Furthermore, Cong et al., using immunolabeling techniques combined with alanine-scanning mutagenesis, pinpointed key IgE-binding amino acids at positions 20, 23, and 27 on β-LG, while positions 26 and 31 were identified as critical IgG-binding residues [47]. Subsequent studies by the same group further identified six key IgE-binding amino acids and five IgG-binding amino acids on α-LA.

Comparative analysis of these studies has confirmed the major allergenic regions within whey proteins. Specifically, the primary IgE-binding regions of α-LA are located at amino acid positions 1–16, 15–26, 62–72, and 93–109, whereas the primary IgE-binding regions of β-LG are found at positions 58–77, 72–78, and 121–134. The identification and characterization of these antigenic epitopes provide valuable insights for assessing the allergenicity of milk proteins and serve as a foundation for developing targeted and efficient strategies to reduce milk protein allergenicity.

## 3. Mechanisms of Cow’s Milk Allergy

**Figure 1:**
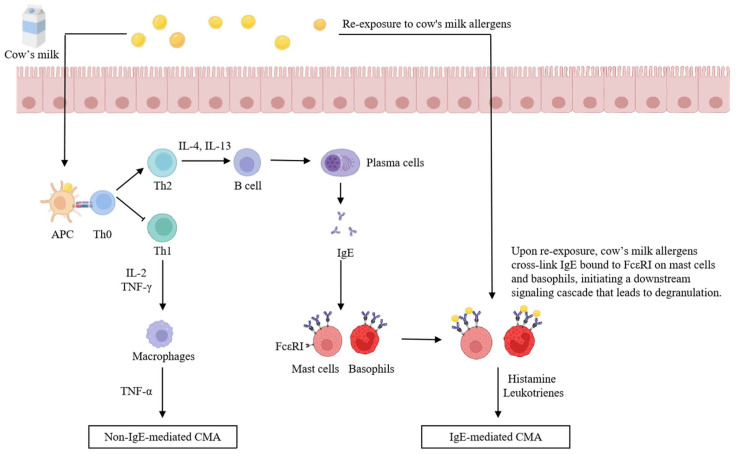
The mechanism of CMA. APC: antigen-presenting cell; IL-2: interleukin-2; IL-4: interleukin-4; IL-13:interleukin-13; IL-5: interleukin-5; Th1: T helper 1 cell;Th2: T helper 2 cell; TNF-γ: -tumor necrosis factor-γ; TNF-α:tumor necrosis factor-α; CMA: cow’s milk allergy; FcεRI: high-affinity immunoglobulin E receptor.

CMA is primarily mediated by non-IgE pathways, though IgE-mediated pathways have been more extensively studied. In the EuroPrevall birth cohort study conducted across nine European countries, a total of 9336 infants were prospectively followed. Among them, 358 children presenting with symptoms suggestive of CMA underwent standardized clinical evaluation. Of the 55 children who were subsequently confirmed to have cow’s milk allergy, approximately 56.3% were classified as having non-IgE-mediated allergy, while 43.7% exhibited IgE-mediated mechanisms [48]. A study in China reported that only 27% of CMA patients exhibit IgE-mediated immune responses [49]. The mechanisms of IgE- and non-IgE-mediated cow’s milk allergy are shown in Figure 1.

### 3.1. IgE-Mediated CMA

IgE-mediated CMA is a rapid-onset hypersensitivity reaction that progresses through two distinct phases. The first phase is the sensitization stage. Upon initial exposure to milk allergens, antigen-presenting cells (APCs) process and present the allergens, leading to the activation of naive T cells (Th0). This results in the differentiation of a limited number of Th1 cells and a predominant expansion of Th2 cells [50]. Th2 cells secrete cytokines such as interleukin-4 (IL-4) and interleukin-13 (IL-13), which induce B cells to undergo class switching and differentiate into plasma cells, thereby producing IgE antibodies [51]. The Fc region of IgE binds to high-affinity IgE receptors (FcεRI) expressed on mast cells and basophils in the bloodstream, rendering the individual sensitized [52]. If further exposure to the allergen is avoided over time, this sensitized state may gradually diminish. The second phase is the effector stage. Upon re-exposure to the same milk allergen, cross-linking of IgE on the surface of mast cells and basophils triggers a signaling cascade, leading to cellular degranulation [53]. This results in the rapid release of bioactive mediators such as histamine and leukotrienes, which provoke local or systemic allergic reactions (Figure 1).

### 3.2. Non-IgE-Mediated CMA

Non-IgE-mediated cow’s milk allergy typically manifests as a delayed hypersensitivity reaction, occurring several hours to days after milk protein ingestion. This process involves the participation of both Th1 and Th2 immune pathways [54]. Upon exposure to milk allergens, APCs process and present the antigens, leading to the activation of Th0. Subsequently, Th1 cells produce interleukin-2 (IL-2) and tumor necrosis factor-γ (TNF-γ), which activate macrophages and elicit an immune response [55] (Figure 1).

## 4. Gut Microbiota Alterations in CMA Patients

The term “gut microbiota” includes all microorganisms, not only bacteria, but also fungi, protists, archaea, and viruses that live in the gastrointestinal tract [56]. Dysbiosis is commonly defined as a decrease in microbial diversity, an absence of beneficial microbes, or the presence of potentially harmful microorganisms [57]. Significant differences in gut microbial composition have been observed between individuals with CMA and healthy controls, particularly in infants and young children. Bunyavanich et al. found that children who outgrew CMA by age eight had gut microbiota enriched in Firmicutes and Clostridiales at 3–6 months, whereas persistent CMA was associated with Bacteroidetes and Enterobacteriaceae dominance [58].

Similarly, Canani et al. found that gut dysbiosis in CMA patients was driven by an overrepresentation of *Bacteroides* and *Ruminococcus* species [59]. Furthermore, dysbiosis associated with both IgE- and non-IgE-mediated CMA exhibited overlapping characteristics, with a significant enrichment of *Bacteroides*. Another study suggested that the ratio of *Enterobacteriaceae* to *Bacteroidaceae* (E/B ratio) could serve as a marker of gut microbiota maturity [60]. In healthy infants, the E/B ratio gradually decreases with age, indicating progressive gut microbiota maturation. However, in CMA infants, an elevated E/B ratio suggests delayed gut microbiota development, which may be a critical predictor of milk allergy. Mauras et al. demonstrated that infants with CMA exhibit lower *Bifidobacterium* and higher *Lachnospiraceae* levels at birth [61]. When the gut microbiota from CMA infants was transplanted into germ-free mice, the lower *Bifidobacterium/Lachnospiraceae* ratio in the infant microbiome promoted a Th2-skewed immune response and aggravated allergic symptoms [61]. Wang et al. further demonstrated that gut microbial dysbiosis associated with non-IgE-mediated cow’s milk protein allergy impairs both the abundance and function of intestinal Treg, thereby disrupting immune tolerance and intestinal homeostasis [62].

These findings highlight a strong correlation between the development of CMA and gut microbiota dysbiosis. Common characteristics of the gut microbiota in CMA patients include an increased ratio of *Enterobacteriaceae* to *Bacteroidaceae*, an enrichment of *Firmicutes*, and a depletion of *Bifidobacterium*. Such dysbiotic changes in the gut microbiota are thought to disrupt immune homeostasis, thereby contributing to the persistence and exacerbation of allergic responses. Therefore, monitoring gut microbiota composition may serve as a valuable approach for predicting and preventing CMA.

## 5. Mechanisms of Probiotic Alleviation of CMA

### 5.1. Application of Probiotics in CMA

Extensive animal studies and clinical research have demonstrated that probiotics supplementation can effectively mitigate the onset and progression of CMA (as shown in Table 1). The application of probiotics in alleviating CMA primarily focuses on maintaining gut microbiota balance, enhancing intestinal barrier function, and regulating the Th1/Th2 immune equilibrium. By administering probiotic therapies and supplements to individuals with CMA, allergic symptoms can be effectively alleviated, contributing to improved immune tolerance and overall management of the condition.

### 5.2. Mechanism of Action of Probiotics in Modulating Cow’s Milk Allergy

#### 5.2.1. Regulation of Intestinal Microbiota

Given the significant differences in the composition and abundance of gut microbiota between allergic and healthy individuals, modulating the gut microbiota represents an effective strategy for managing CMA. Under conditions of nutrient limitation, probiotics compete with harmful bacteria in the intestinal wall for colonization sites and essential nutrients [82]. Additionally, probiotics can utilize their own digestive enzymes to break down and metabolize incompletely hydrolyzed dietary components in the gastrointestinal tract [83]. Under normal physiological conditions, *Lacticaseibacillus* species are predominant in the distal small intestine and proximal colon, while *Bifidobacterium* species dominate the distal colon. Therefore, supplementation with *Lacticaseibacillus* or *Bifidobacterium* can effectively reduce microbial competition for nutrients [84]. Moreover, Bifidobacterium has been shown to enhance mucin secretion by intestinal epithelial cells and regulate gastrointestinal hormone levels. The adhesion of probiotics to mucus and intestinal epithelial cells not only provides a competitive advantage but also prevents pathogenic bacteria from adhering to intestinal epithelial cells [85]. For instance, *Bifidobacterium*, through its adhesion to teichoic acid in the bacterial cell wall, facilitates the synthesis of extracellular glycosidases within intestinal epithelial cells, thereby degrading potential pathogens and forming a protective intestinal barrier, effectively preventing pathogenic invasion, adhesion, and colonization [86,87].

Numerous clinical studies have demonstrated that probiotic supplementation is one of the most direct methods for regulating host gut microbiota, playing a significant role in alleviating CMA symptoms. One trial using EHCF supplemented with LGG in 19 CMA infants showed increased butyrate levels and enrichment of beneficial genera such as *Blautia* and *Roseburia* in tolerant individuals, indicating improved oral tolerance [59]. Mennini et al. found that long-term colonization of *Bifidobacterium longum* subsp. *infantis* M-63 increased the abundance of *Akkermansia* and *Ruminococcus* in the gut of CMA infants, promoting beneficial gut microbiota regulation. The biological rationale for using *Bifidobacterium longum* subsp. *infantis* M-63 in the treatment of CMA is therefore well-supported [88]. Another intervention using *Bifidobacterium* TMC3115 reduced allergy scores and enhanced anti-inflammatory responses in CMA infants, altering microbial composition toward beneficial phyla [67]. A study involving CMA infants aged 0–12 months demonstrated that daily administration of LGG for four consecutive weeks significantly improved allergic symptoms such as hematochezia, diarrhea, and bloating [65]. However, its effects on abdominal pain, constipation, and skin inflammation were less pronounced.

In summary, probiotic intake positively regulates gut microbiota composition, enhances immune tolerance, and improves allergic symptoms in CMA infants. While many probiotics have demonstrated anti-allergic properties, the increasing availability of multi-strain probiotic formulations necessitates rigorous clinical evaluation of their colonization capacity and persistence within the gut before widespread clinical application. A deeper understanding of strain-specific effects will be critical for optimizing therapeutic outcomes in CMA management.

#### 5.2.2. Enhancement of the Intestinal Barrier

**Figure 2:**
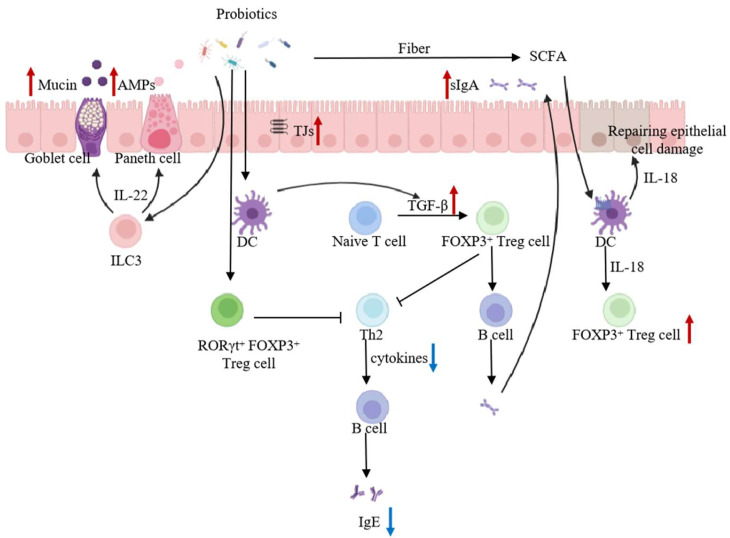
Potential mechanisms of probiotics to alleviate CMA. DC: dendritic cell; SCFA: short-chain fatty acid; AMPs: antimicrobial peptides; Treg: regulatory T cell; Th2: T helper 2 cell, IL-18: interleukin-18; IL-22: interleukin-22; TJs: tight Junctions; TGF-β: transforming growth factor-β; CMA: cow’s milk allergy; ↑, up-regulated; ↓, down-regulated.

The intestinal barrier is a dynamic system composed of four interrelated components: the mechanical, chemical, immune, and microbial barriers. These barriers work synergistically to defend against pathogens and prevent the penetration and absorption of allergens. The mechanical barrier consists of intestinal epithelial cells and their tight junctions, which selectively regulate the transport of substances, preventing allergens and inflammatory mediators from infiltrating the intestinal mucosa [89]. Probiotics can enhance the integrity of the mucosal barrier by upregulating the expression of tight junction proteins in intestinal epithelial cells [90]. The chemical barrier primarily comprises digestive secretions from intestinal goblet cells and antimicrobial compounds produced by probiotics. Studies have shown that *Clostridium* species and other commensal bacteria interact with group 3 innate lymphoid cells (ILC3s) to stimulate the production of interleukin-22 (IL-22), which in turn induces Paneth cells to release antimicrobial peptides and promotes goblet cells to secrete mucus [91] (Figure 2). This process helps regulate the uptake of milk allergens. The microbial barrier is formed by the gut microbiota, which constitutes a self-regulating and interdependent ecosystem. The balance of this microbial community plays a crucial role in maintaining the intestinal barrier. Probiotics, through competitive exclusion and the secretion of metabolic byproducts, contribute to gut barrier function, not only enhancing immune responses but also further degrading allergenic proteins [92,93]. In infants, limited enzymatic capacity and an underdeveloped intestinal barrier allow partially digested milk proteins to enter the systemic circulation more easily [94,95]. Gut dysbiosis further compromises barrier integrity, enabling greater allergen translocation and intensifying allergic responses [96].

Probiotic colonization can form a protective layer on the intestinal mucosa, restoring and reinforcing barrier function while simultaneously promoting microbial homeostasis. This, in turn, strengthens the immune barrier and enhances oral tolerance. Therefore, the ability of probiotics to modulate intestinal barrier function may be a key mechanism in preventing the entry of milk allergens and mitigating allergic reactions.

#### 5.2.3. Promotion of Intestinal Mucosal Immunity

Studies have shown that CMA in infants is closely linked to the immaturity of the intestinal mucosal immune system, particularly the low secretion of secretory IgA (SIgA), which weakens the defense against milk allergens [97,98]. Probiotics can enhance mucosal immunity by stimulating immune cells and promoting SIgA production. SIgA binds to allergens in the intestinal mucus layer, limiting their absorption and preventing systemic sensitization [99]. *Lactobacillus acidophilus* LaVK2 and *Bifidobacterium* BbVK3 increased Th1 cytokines while reducing Th2 cytokine IL-4 in whey-allergic mice, suggesting that probiotics alleviate allergic responses by enhancing Th1-type immunity and suppressing excessive Th2-mediated reactions, thereby restoring the Th1/Th2 immune balance [83]. Neau et al. investigated the effects of *Lacticaseibacillus salivarius* LA307, *Bifidobacterium longum* subsp. *infantis* LA308, and *Lacticaseibacillus rhamnosus* LA305 in mice allergic to β-lactoglobulin. The study found that *Lacticaseibacillus salivarius* LA307 exhibited strong immunosuppressive effects by inhibiting both Th1 and Th2 responses and potentially inducing regulatory T (Treg) cells. *Bifidobacterium. longum* subsp. *infantis* LA308 promoted Th1 cytokine production while inhibiting Th2 cell proliferation, whereas *Lacticaseibacillus rhamnosus* LA305 modulated and enhanced Th1 responses [70]. These findings indicate that these probiotic strains contribute to the restoration of Th1/Th2 immune balance and alleviate CMA symptoms. Further research has demonstrated that the gut microbiota induces a specific subset of CD4⁺ FoxP3⁺ Treg cells that also express RORγt⁺, the key transcription factor of Th17 cells. This novel subset of Treg, referred to as Tr3 cells, plays a crucial role in immune modulation [100]. To further explore their function, researchers administered *Lacticaseibacillus casei* BL23 to CMA-induced mice and found that it induced both local and systemic FoxP3⁺ RORγt⁺ Tr3 cells, which contributed to the immune-enhancing effects of *Lacticaseibacillus casei* BL23 against CMA [71]. Sardecka-Milewska et al. reported that increased FoxP3 mRNA expression accelerates the acquisition of tolerance in CMA infants. Moreover, FoxP3⁺ Treg cells promote the secretion of IgA and IgG by B cells while inhibiting Th2 cells and IgE production, ultimately alleviating allergic responses to cow’s milk proteins [101] (Figure 2).

In addition, probiotic metabolites, particularly short-chain fatty acids (SCFAs), can directly interact with host cells and pathogens, transmitting immunomodulatory signals. SCFAs activate dendritic cells (DCs) through G-protein-coupled receptors, leading to the production of interleukin-18 (IL-18), which not only repairs epithelial damage but also promotes the proliferation of FoxP3⁺ Treg cells [102]. FoxP3⁺ Tregs exert anti-inflammatory effects by secreting interleukin-10 (IL-10), interleukin-35 (IL-35), and transforming growth factor-β (TGF-β), thereby maintaining oral tolerance to cow’s milk proteins [103]. Probiotics and their metabolic products alleviate symptoms of CMA by inducing the production of Treg, regulating the Th1/Th2 balance, and directing the class-switch recombination of B cells (Figure 2).

#### 5.2.4. Decomposition of Cow’s Milk Allergenic Proteins

Probiotics can reduce the allergenicity of cow’s milk proteins by enzymatically degrading milk allergens. Zhao et al. reported that Clostridium butyricum strain Z816 exhibited exceptional degradation capabilities against the major milk allergen β-LG, significantly reducing its allergenicity and alleviating CMA symptoms [104]. The degradation of β-LG was attributed to protease production by *Clostridium tyrobutyricum* Z816, as well as enhanced cell permeability, which facilitated improved substrate-protease interactions, thereby increasing degradation efficiency. Micael et al. simulated gastrointestinal digestion in vitro and found that pre-hydrolysis of β-LG by *Lacticaseibacillus salivarius* LA307 and *Lactobacillus delbrueckii subsp*. *bulgaricus* CRL 454 significantly enhanced its digestibility, thereby mitigating allergic responses [105]. Additionally, probiotics can degrade large allergenic proteins through fermentation, disrupting their antigenic epitopes and reducing their immunogenicity [106]. For instance, fermentation with *Lacticaseibacillus casei* LcY alone effectively reduced the immunoreactivity of α-LA and β-LG, and their allergenicity was further diminished after simulated digestion [107]. Similarly, co-fermentation with Lactobacillus helveticus and Streptococcus thermophilus significantly decreased the antigenicity of α-LA and β-LG, with observed synergistic effects between the strains [80,108]. Regarding caseins, Biscola et al. demonstrated that during the fermentation of ultra-high temperature (UHT)-treated skim milk, *Enterococcus faecium* VB63F produced proteases capable of effectively hydrolyzing the major milk allergens αs1-casein, αs2-casein, and β-casein, thereby reducing their allergenicity [107].

## 6. Conclusions and Prospects

The application of probiotics in modulating the gut microbiota–immune axis to alleviate CMA has emerged as a significant research focus. Current studies indicate that probiotics contribute to restoring gut microbial balance, enhancing intestinal barrier function, and regulating immune responses, thereby playing a crucial role in alleviating allergic reactions. However, the precise mechanisms by which probiotics exert their anti-allergic effects remain under investigation, necessitating further in vitro and in vivo studies to establish their efficacy and safety.

One promising area of research involves the incorporation of probiotics into hypoallergenic infant formula to enhance immune tolerance in CMA patients. While hydrolyzed infant formulas—either casein-based or whey-based—are widely used to manage CMA, residual allergenic epitopes in some formulations may still trigger allergic responses. Recent advancements have explored the combination of enzymatic hydrolysis with probiotic supplementation to create hypoallergenic milk protein hydrolysates with broader applications. Notably, the addition of LGG to extensively hydrolyzed casein EHCF has demonstrated potential in accelerating immune tolerance acquisition, reducing the incidence of allergic dermatitis, and mitigating intestinal inflammation. Nonetheless, concerns remain regarding the long-term safety and effectiveness of probiotic-enhanced formulas. Current challenges include variability in probiotic strain specificity, dosage, and treatment duration, which hinder the development of standardized clinical protocols. Future studies should clarify strain-specific immunomodulatory mechanisms, optimize supplementation regimens, and assess the long-term impact on immune development. Additionally, a more refined understanding of how different probiotic strains interact with casein and whey protein hydrolysates is needed to optimize hypoallergenic formula formulations.

In conclusion, probiotics hold significant promise as a complementary approach for managing CMA, yet their application requires deeper mechanistic insights and robust clinical validation. Future research should focus on optimizing probiotic-based interventions, determining individualized treatment strategies based on genetic and microbiota profiles, and ensuring the long-term safety of probiotic-enriched hypoallergenic formulas. Establishing standardized protocols for probiotic supplementation will be critical for integrating these promising microbial therapies into mainstream CMA management.

## Figures and Tables

**Table 1:** Application of probiotics in alleviation of CMA.

Species	Allergen	Probiotics	Treatment	Results	Reference
Infant	Cow’s milk	*Lacticaseibacillus rhamnosus* GG (LGG)	Infants aged 0–12 months with CMA were administered an extensively hydrolyzed casein formula (EHCF) supplemented with LGG, and were followed up for a period of 36 months.	Reduce the incidence of other allergic manifestations and accelerate the development of oral tolerance.	[63]
Infant	Cow’s milk	LGG	Infants aged 0–12 months with CMA were administered an EHCF supplemented with LGG for a period of 36 months.	Improving infant gut microbial composition, diversity, and metabolites to promote tolerance	[64]
Infant	Cow’s milk protein	LGG	Infants aged 0–12 months with cow’s milk protein allergy were administered LGG alongside a milk-free diet for a period of 4 weeks.	Improving bloody stools, diarrhoea, restlessness, and bloating.	[65]
Infant	Cow’s milk protein	*Bifidobacteria*	Infants aged 6–12 months with cow’s milk protein allergy were administered *Bifidobacteria* alongside a milk-free diet for a period of 45 days.	Reduce naive and activated CD4+ T cells as well as degranulated basophilic granulocytes.	[66]
Infant	Cow’s milk protein	*Bifidobacterium bifidum* TMC3115	Infants aged 0–12 months with cow’s milk protein allergy were administered *Bifidobacterium bifidum* TMC3115 for a period of 6 months.	Reduce allergy scores, enhance anti-inflammatory responses, decrease serum IgE levels, increase IgG2 levels, and regulate the gut microbiota.	[67]
Mice	β-LG	*Lactobacillus delbrueckii* subsp *bulgaricus* CRL 656 (H656)	After hydrolysis of β-LG with H656, BALB/c mice were gavaged.	After hydrolyzing β-LG with H656, the allergic response induced by β-LG was suppressed by increasing the secretion of IL-6, IL-10, and IFN-γ, reducing IL-4 levels, improving intestinal mucosal damage, and decreasing leukocyte infiltration.	[68]
Mice	α-Caseins and β-LG	yogurt beverage (*Lactiplantibacillus plantarum* and *Bifidobacterium animalis* subsp. *lactis)*	Administer the yogurt beverage to BALB/c mice via gavage for a period of 4 weeks	Enhanced the secretion of IL-10, TGF-β, and IgA, while reducing the levels of IL-4, IgE, and IgG1.	[69]
Mice	β-LG	*Lactobacillus salivarius LA307*, *Bifidobacterium longum* subsp. *infantis* LA308, *Lacticaseibacillus rhamnosus* LA305	Administer probiotics to BALB/c mice via gavage for a period of 6 weeks.	*Lactobacillus salivarius* LA307 blocked Th1 and Th2 responses; *Bifidobacterium longum* subsp. *infantis* LA308 induced a pro-Th1 response; *Lacticaseibacillus rhamnosus* LA305 induced both a pro-Th1 and an immunoregulatory response.	[70]
Mice	Cow’s milk	*Lacticaseibacillus casei* BL23	Administer *Lacticaseibacillus casei* BL23 to BALB/cJ mice via gavage continuously for 5 days.	Induces both local and systemic Foxp3^+^ RORγt^+^ type 3 regulatory T cells (Tr3).	[71]
Mice	α-Caseins and β-LG	Fermented whey (*Streptococcus salivarius* subsp. *thermophilus* 2 K, *Lactobacillus delbrueckii* subsp. *bulgaricus* BK—FW whey, *S. thermophilus* 2 K, *L. bulgaricus* BK, *Lactiplantibacillus plantarum* W42, and *Bifidobacterium animalis* ssp. *lactis* Bi30—FW-LB)	Administer the fermented whey to BALB/c mice via gavage for a period of 4 weeks	Altered the Th1/Th2 balance towards a Th1 response, enhanced the secretion of IL-10 and TGF-β, and reduced the levels of allergy markers.	[72]
Mice	skimmed milk	Fermented milk beverage (*L. plantarum* DPUL-F20, *L. paracasei* DPUL-F29, *L. bulgaricus* DPUL-F36 and *S. thermophilus* BD0453)	Administer the Fermented milk beverage to BALB/c mice via gavage for a period of 7 weeks	Regulated the Th1/Th2 and Th17/Treg immune balance, reduced the levels of total IgG, total IgG1, and total IgE antibodies, serum mast cell protease, and plasma histamine levels, and modulated the composition of the gut microbiota.	[73]
Mice	β-LG	*Clostridium butyricum* CGMCC0313-1	Administer *Clostridium butyricum* CGMCC0313-1 to BALB/cJ mice via gavage for a period of 3 weeks.	Improved intestinal allergic reaction symptoms. Increased levels of sIgA and CD4^+^ CD25^+^ Foxp3^+^ Treg cells. Reversed the imbalance between Th1/Th2 and Th17/Treg.	[74]
Rat	Whey protein	*Lactiplantibacillus plantarum* DPUL-F232	Administer *Lactiplantibacillus plantarum* DPUL-F232 to SD rat via gavage.	Alleviated allergic symptoms, reduced intestinal inflammation, and lowered serum antibody and histamine levels in rats; regulated the Th1/Th2 balance, promoted the secretion of IL-10, and inhibited mast cell degranulation. Upregulated the expression of tight junction proteins to restore the integrity of the intestinal barrier; modulated the gut microbiota and its metabolic products to alleviate allergies.	[75,76]
In Vitro	Cow’s milk protein	*Lacticaseibacillus paracasei* XJ-003	Fermented	Significantly reduced the antigenicity of milk proteins.	[77]
In Vitro	Cow’s milk and butter milk	Lactic acid bacteria and *Bifidobacterium*	Fermented	Fermentation with *L. casei* LcY bacteria significantly reduced the immunoreactivity of BLG, α-CN, β-CN, κ-CN, and raw milk by 98%, 96%, 89%, 75%, and 93%, respectively. Similarly, fermentation with *L. delbrueckii* ssp. *bulgaricus* 151 resulted in reductions in the immunoreactivity of the same proteins by 98%, 95%, 90%, 71%, and 89%. A significant reduction in IgE reactivity was only observed in the products fermented by both bacterial strains together.	[78]
In Vitro	α-LA and β-LG	Lactic acid bacteria	Fermented	Reduced the allergenic potential of α-LA and β-LG	[79]
In Vitro	α-LA and β-LG	*Lactobacillus helveticus* and *Streptococcus thermophilus*	Fermented	Reduced the allergenic potential of α-LA and β-LG	[80]
In Vitro	α-LA and β-LG	*Streptococcus thermophilus* and *Lactobacillus delbrueckii* subsp. *bulgaricus.*	Fermented	Block allergenic epitopes, produce bioactive peptides; modulate the immune system.	[81]

## Data Availability

The original contributions presented in the study are included in the article. Further inquiries can be directed to the corresponding authors.
